# Market Chickens as a Source of Antibiotic-Resistant *Escherichia coli* in a Peri-Urban Community in Lima, Peru

**DOI:** 10.3389/fmicb.2021.635871

**Published:** 2021-03-02

**Authors:** Matthew Murray, Guillermo Salvatierra, Alejandra Dávila-Barclay, Brenda Ayzanoa, Camila Castillo-Vilcahuaman, Michelle Huang, Mónica J. Pajuelo, Andrés G. Lescano, Lilia Cabrera, Maritza Calderón, Douglas E. Berg, Robert H. Gilman, Pablo Tsukayama

**Affiliations:** ^1^Johns Hopkins School of Medicine, Baltimore, MD, United States; ^2^Laboratorio de Genómica Microbiana, Facultad de Ciencias y Filosofía, Universidad Peruana Cayetano Heredia, Lima, Peru; ^3^Emerge, Emerging Diseases and Climate Change Research Unit, School of Public Health and Administration, Universidad Peruana Cayetano Heredia, Lima, Peru; ^4^Johns Hopkins Bloomberg School of Public Health, Baltimore, MD, United States; ^5^Laboratorio de Microbiología Molecular, Facultad de Ciencias y Filosofía, Universidad Peruana Cayetano Heredia, Lima, Peru; ^6^Asociación Benéfica PRISMA, Lima, Peru; ^7^Laboratorios de Enfermedades Infecciosas, Laboratorios de Investigación y Desarrollo, Universidad Peruana Cayetano Heredia, Lima, Peru; ^8^Department of Medicine, University of California, San Diego, San Diego, CA, United States; ^9^Instituto de Medicina Tropical Alexander von Humboldt, Universidad Peruana Cayetano Heredia, Lima, Peru; ^10^Wellcome Sanger Institute, Hinxton, United Kingdom

**Keywords:** AMR, genomics, LMIC, poultry, *Escherichia coli*, one health, WGS, Peru

## Abstract

The widespread and poorly regulated use of antibiotics in animal production in low- and middle-income countries (LMICs) is increasingly associated with the emergence and dissemination of antibiotic resistance genes (ARGs) in retail animal products. Here, we compared *Escherichia coli* from chickens and humans with varying levels of exposure to chicken meat in a low-income community in the southern outskirts of Lima, Peru. We hypothesize that current practices in local poultry production result in highly resistant commensal bacteria in chickens that can potentially colonize the human gut. *E. coli* was isolated from cloacal swabs of non-organic (*n* = 41) and organic chickens (*n* = 20), as well as from stools of market chicken vendors (*n* = 23), non-vendors (*n* = 48), and babies (*n* = 60). 315 *E. coli* isolates from humans (*n* = 150) and chickens (*n* = 165) were identified, with chickens showing higher rates of multidrug-resistant and extended-spectrum beta-lactamase phenotypes. Non-organic chicken isolates were more resistant to most antibiotics tested than human isolates, while organic chicken isolates were susceptible to most antibiotics. Whole-genome sequencing of 118 isolates identified shared phylogroups between human and animal populations and 604 ARG hits across genomes. Resistance to florfenicol (an antibiotic commonly used as a growth promoter in poultry but not approved for human use) was higher in chicken vendors compared to other human groups. Isolates from non-organic chickens contained genes conferring resistance to clinically relevant antibiotics, including *mcr-1* for colistin resistance, *blaCTX-M* ESBLs, and *blaKPC-3* carbapenemase. Our findings suggest that *E. coli* strains from market chickens are a potential source of ARGs that can be transmitted to human commensals.

## Introduction

Antimicrobial resistance (AMR) in human pathogens has become a major global health threat (O’Neill, 2014; World Health Organization [WHO], 2017b), with bacterial infections increasingly failing to first-line and “last-resort” antibiotic therapies. Decades of widespread antibiotic use in medicine and agriculture (Silbergeld et al., 2008) have resulted in the emergence and spread of various resistance determinants in microbial populations. In particular, the increasing demand for animal protein has led to a dramatic modernization of agriculture, including the regular use of antibiotics in feed to promote animal growth in addition to their therapeutic use. At low (sub-inhibitory) but constant dosages, antibiotics serve as growth promoters by reducing the levels of pathogenic strains and altering the microbiota to allow the host for more nutrient uptake (Evans and Wegener, 2003). This selective pressure has dramatically increased the rate of resistance to various drugs in the microbiota of farm animals, including commensals and pathogens alike (Woolhouse et al., 2015; Robinson et al., 2016; Liu et al., 2018; Nadimpalli et al., 2018; Van Boeckel et al., 2019). Resistant strains can be transmitted from animals to humans through meat consumption, direct animal contact, and exposure to environmental runoff (Hoelzer et al., 2017). Furthermore, horizontal gene transfer can enable the rapid exchange of resistance determinants between different bacterial lineages across hosts and environments (Marshall and Levy, 2011; Woolhouse et al., 2015). Because most antibiotic resistance genes (ARGs) are found in bacteria isolated from both humans and animals, the direction of transfer of most such genes and resistant organisms can be difficult to demonstrate.

We previously surveyed the antibiotic resistomes in the guts of healthy adults in a peri-urban community south of Lima and found high diversity and abundance of genes encoding resistance to amphenicol antibiotics (Pehrsson et al., 2016). A recent study in Cambodia compared *E. coli* isolates from humans, meat, and fish and found moderate levels of amphenicol resistance in human isolates (Nadimpalli et al., 2019). Although used widely until the 1980s, chloramphenicol is now rarely prescribed in human medicine in Peru and is banned from food animal production since 2013 (Diario Oficial El Peruano, 2013). However, florfenicol (a fluorinated thiamphenicol analog) is widely employed in broiler farming therapeutically and as a growth promoter and available in various commercial feed premixes (FAO, 2014). This has led to the hypothesis that amphenicol resistance in human commensals did not emerge from clinical use, but in food animal populations due to extensive veterinary use of chloramphenicol, florfenicol, and other related compounds. Chickens, most of which now are grown under local intensive farming systems, provide the primary source of animal protein for the Peruvian population (World Bank Group, 2017). Average per capita consumption was estimated at 49.5 kg in 2018, and up to 80.5 kg per person per year in the capital of Lima (Ministerio de Agricultura y Riego [MINAGRI], 2018).

We hypothesize that current practices in poultry production and handling in LMICs result in highly resistant chicken commensals that can potentially colonize the human gut. To test this, we assessed the distribution of resistant *E. coli* and associated ARGs in market chickens, chickens grown without antibiotics (organic chickens), and residents from a low-income, peri-urban community in Lima, with varying levels of exposure to poultry.

## Materials and Methods

### Study Site

Local market stalls in Villa El Salvador (VES) and its neighboring district, San Juan de Miraflores (SJM) in southern Lima, were visited to purchase whole chickens. Human fecal samples were collected from the community surrounding the VES market (see Supplementary Table 1). These neighboring districts share similar demographic characteristics and contain various urban informal settlements (Instituto Nacional de Estadística e Informática [INEI], 2017). Informal housing arrangements, lack of running water, and inadequate sanitation in most households make these sites representative of peri-urban settlements in other LMICs, which are considered hotspots for AMR (Nadimpalli et al., 2020). We also collected laying hens’ samples from an organic free-range farm in Vegueta (VEG), located approximately 150 km north of Lima.

### Samples

#### Humans

Fecal samples were collected in March 2018 from three resident groups in the VES community: chicken vendors (*n* = 23) working in the markets where chickens were purchased, babies (*n* = 60) between 1 and 24 months old from an ongoing cohort study in the community, and non-vendor adults (*n* = 48). Fresh feces were collected by individuals and legal guardians as instructed. Fecal samples were swabbed and placed vials with Cary-Blair transport medium, stored at 4°C, and transferred to the laboratory for further processing. Ethical approval was obtained from Institutional Review Boards at Universidad Peruana Cayetano Heredia and Asociación Benéfica Prisma.

#### Market (Non-organic) Chickens

Forty-one recently slaughtered whole chickens were purchased in 14 market stalls of VES and SJM from March to April of 2018. Whole chickens and market stands were selected by convenience. We have no information on the exact rearing conditions or origin of these chickens. However, almost all of the chicken meat sold in Lima originates from conventional local production systems that heavily rely on routine antibiotic use as a standard industry practice. Chickens were taken to field laboratories for the collection of intestinal contents. Cloacal and intestinal swabs were put in sterile tubes with saline solution and transferred within 2 h to the laboratory for bacterial culture.

#### Organic Laying Hens

Cloacal swabs from 20 laying hens from the sole Certified Humane^®^ (Humane Farm Animal Care [HFAC], 2018) organic free-range farm in Lima were obtained in May of 2019 to have a set of isolates originating from poultry raised without antibiotics as a comparison group to the market chickens. Cloacal swabs were put in sterile tubes with Cary-Blair transport medium, stored at 4°C, and transferred to the laboratory for processing.

### Culture and Isolation

Samples were streaked in CHROMagar Orientation Media (CHROMagar Microbiology, Paris, France) for rapid differentiation and presumptive identification of *E. coli*. Up to 3–5 dark pink to red colonies indicative of *E. coli* were re-streaked to MacConkey agar (Becton Dickinson, Heidelberg, Germany) for lactose fermentation confirmation and then selected for species confirmation with a conventional biochemical profiling panel (Garrity et al., 2005). Those confirmed as *E. coli* (*n* = 315) were included in the study and stored in Tryptic soy broth (TSB, Becton Dickinson) with glycerol at −20°C until DNA extraction.

### Antibiotic Susceptibility Testing

Disk diffusion tests were performed with CLSI 2018 standards, using susceptible, intermediate, and resistant definitions for *Enterobacteriaceae* (Clinical and Laboratory Standards Institute (CLSI), 2018). A total of 18 antibiotics were used (see Supplementary Table 2). Extended-spectrum β-lactamase (ESBL) activity was detected using the cefotaxime-ceftazidime-cefepime-aztreonam with amoxicillin with clavulanic acid test, according to EUCAST standards (The European Committee on Antimicrobial Susceptibility Testing [EUCAST], 2017). We interpreted florfenicol susceptibility using chloramphenicol’s CLSI breakpoints as there are no approved cut-off values for *E. coli* (White et al., 2000; Clinical and Laboratory Standards Institute (CLSI), 2018). We did not report on colistin phenotypic resistance due to the lack of recommended cut-off values for colistin disk diffusion testing (Ezadi et al., 2018). A multidrug-resistant drug isolate was defined as expressing phenotypic resistance to three or more antibiotic classes (Magiorakos et al., 2012).

### DNA Extraction and WGS

DNA was extracted from 1 ml TSB culture using the GeneJet Genomic DNA purification kit (Thermo Fisher Scientific, Waltham, MA, United States) following the manufacturer’s instructions. DNA was eluted in 200 μl Tris-EDTA buffer and quantified using the Qubit dsDNA BR Kit (Thermo Fisher Scientific). We selected a subset of 118 isolates for WGS on the Illumina MiSeq platform. We randomized isolate selection within each study group to include representative drug susceptibility patterns. Libraries were prepared from 1 ng gDNA with the Nextera XT kit (Illumina, San Diego, CA, United States). Batches of 24 libraries were indexed and sequenced with MiSeq v3 sequencing kits to generate 300 bp paired-end reads and yield a mean of 84x genome coverage (minimum 17x, maximum 163x). Raw Illumina reads were uploaded to GenBank under BioProject PRJNA633873.

### Genomic and Phylogenetic Analyses

Raw reads were assessed with FastQC v0.11.9, trimmed with Trimmomatic v0.36.6 (Bolger et al., 2014), assembled with SPAdes v.3.10.0 (Bankevich et al., 2012), and annotated with Prokka v1.5 (Seemann, 2014). MLST was determined from *de novo* assemblies using the CGE pipeline (Thomsen et al., 2016) based on the Enterobase scheme^1^ accessed through PubMLST^2^. ARGs were annotated by querying assemblies against the CARD database (Alcock et al., 2020) at >90% identity. We clustered ARG-containing contigs with CD-HIT (Li and Godzik, 2006) at an 80% similarity threshold over the contig’s length. Plasmid typing was done using the PlasmidFinder database (Carattoli et al., 2014) and BLAST (Ye et al., 2006) to identify assemblies containing an Inc reference gene, with a threshold of 90% identity and E-value <1e-35. Prokka-annotated assemblies were used as input for Roary v3.13.0 (Page et al., 2015) to determine the pangenome and perform a core gene alignment of all sequenced isolates using blastp identity threshold of 95%. Variable positions were extracted from an alignment of 2,233 core genes (2,252,390 bp) and used to build a maximum-likelihood phylogenetic tree with RAxML v8.2.4 (Stamatakis, 2014) with the general time-reversible (GTR) substitution model and gamma correction for rate heterogeneity. SNP-dists v0.7.0 was used to build a pairwise SNP distance matrix from the pangenome alignment. A published genome of *Escherichia fergusonii* (Manninger et al., 2016) was used to root the phylogenetic tree. CLC Genomics Workbench v20.0 (QIAGEN Bioinformatics) was used to visualize and annotate the tree.

### Statistical Analysis

The proportion of resistant isolates was tabulated for each sample type. Comparisons of proportions were evaluated using the Chi-square test or Fisher’s exact test as appropriate. Data management and statistical analysis were performed with a confidence level of 95% using STATA 16 (StataCorp, College Station, TX, United States) and R (v3.5.2).

## Results

### Antimicrobial Susceptibility

#### Chickens

**FIGURE 1:**
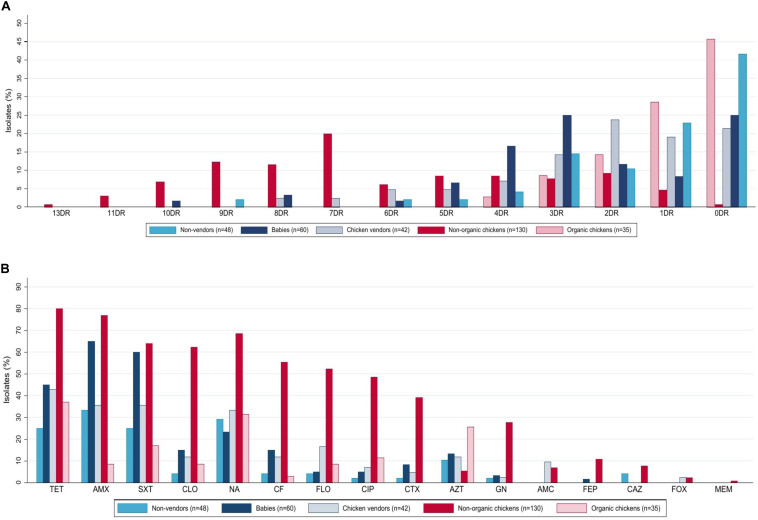
Phenotypic antibiotic resistance of 315 *E. coli* isolates from humans and chickens in Lima, Peru. **(A)** Isolates were grouped by number of antibiotics to which they were resistant based on disk-diffusion assays. **(B)** Based on resistance to 16 antibiotics. **(A)** Percentage of resistances to different drugs, DR, drug resistance. **(B)** Resistance patterns to different antibiotics, TET, tetracycline; AMX, amoxicillin; SXT, trimethoprim/sulfamethoxazole; NA, nalidixic acid; CLO, chloramphenicol; CF, cefalotin; FLO, florfenicol; CIP, ciprofloxacin; CTX, cefotaxime; AZT, azithromycin; GN, gentamicin; AMC, Amoxicillin with Clavulanic Acid; FEP, cefepime; CAZ, ceftazidime; FOX, cefoxitin; MEM, meropenem.

*Escherichia coli* isolates were obtained from market (non-organic) (*n* = 130) and organic (*n* = 35) chickens. Multidrug-resistant (MDR) rates were higher in non-organic animals (76.9 vs. 11.4%, *p* < 0.001, Chi-square test). Only the non-organic chicken isolates were ESBL producers (39.2%, *n* = 51), and presented resistance to at least five antibiotic families (46.2%, *n* = 60), including chloramphenicol (62.3%, *n* = 81), florfenicol (52.3%, *n* = 68), and meropenem (0.8%, *n* = 1). These isolates presented the highest resistance levels to almost every antimicrobial tested. In contrast, the organic chicken isolates were susceptible gentamicin, amoxicillin with clavulanic acid, cefotaxime, cefepime, ceftazidime, and cefoxitin (Figure 1). A comparison of resistance rates is detailed in Table 1.

**TABLE 1:** Resistance profiles and bivariate analysis of *E. coli* isolates from chickens and humans.

Results	Humans							Chickens				Total (n=315)	p^e^
			
	Total (n=150)	N (%)			p^a^	p^b^	p^c^	Total (n=165)	N (%)		p^d^		
		Non-vendor adults (n=48)	Babies (n=60)	Vendors (n=42)					Non-organic (n=130)	Organic (n=35)			
**Multidrug-resistance**													
Yes	56 (37.3)	11 (22.9)	29 (48.3)	16 (38.1)	0.007	0.305	0.117	104 (63)	100 (76.9)	4 (11.4)	<0.001	160 (50.8)	<0.001
**ESBL**													
Yes	6 (4)	1 (2.1)	4 (6.7)	1 (2.4)	0.379*	0.646*	1.000*	51 (30.9)	51 (39.2)	0 (0)	<0.001	57 (18.1)	<0.001
**Amphenicols**													
Cloramphenicol	16 (10.7)	2 (4.2)	9 (15)	5 (11.9)	0.107*	0.655	0.245*	84 (50.9)	81 (62.3)	3 (8.6)	<0.001	100 (31.8)	<0.001
Florfenicol	12 (8)	2 (4.2)	3 (5)	7 (16.7)	1.000*	0.087*	0.077*	71 (43.1)	68 (52.3)	3 (8.6)	<0.001	83 (26.4)	<0.001
**Tetracyclines**													
Tetracycline	57 (38)	12 (25)	27 (45)	18 (42.9)	0.032	0.830	0.073	117 (70.9)	104 (80)	13 (37.1)	<0.001	174 (55.2)	<0.001
**Sulfonamides**													
Trimethoprim/sulfamethoxazole	63 (42)	12 (25)	36 (60)	15 (35.7)	<0.001	0.016	0.268	89 (53.9)	83 (63.9)	6 (17.1)	<0.001	152 (48.3)	0.034
**Aminoglycosides**													
Gentamicin	4 (2.7)	1 (2.1)	2 (3.3)	1 (2.4)	1.000*	1.000*	1.000*	36 (21.8)	36 (27.7)	0 (0)	<0.001	40 (12.7)	<0.001
**Macrolides**													
Azithromycin	18 (12)	5 (10.4)	8 (13.3)	5 (11.9)	0.643	0.831	1.000*	16 (9.7)	7 (5.4)	9 (25.7)	<0.001	34 (10.8)	0.511
**Penicillins**													
Amoxicillin	70 (46.7)	16 (33.3)	39 (65)	15 (35.7)	0.001	0.004	0.813	103 (62.4)	100 (76.9)	3 (8.6)	<0.001	173 (54.9)	0.005
Amoxicillin with Clavulanic Acid	4 (2.7)	0 (0)	0 (0)	4 (9.5)	N.A.	0.026*	0.044*	9 (5.5)	9 (6.9)	0 (0)	0.207*	13 (4.1)	0.214
**Cephalosporins**													
Cefalotin	16 (10.7)	2 (4.2)	9 (15)	5 (11.9)	0.107*	0.655	0.245*	73 (44.2)	72 (55.4)	1 (2.9)	<0.001	89 (28.3)	<0.001
Cefotaxime	8 (5.3)	1 (2.1)	5 (8.3)	2 (4.8)	0.223*	0.697*	0.597*	51 (30.9)	51 (39.2)	0 (0)	<0.001	59 (18.7)	<0.001
Cefepime	1 (0.7)	0(0)	1 (1.7)	0 (0)	1.000*	1.000*	N.A.	14 (8.5)	14 (10.8)	0 (0)	0.042*	15 (4.7)	0.001
Ceftazidime	2 (1.3)	2 (4.2)	0(0)	0(0)	0.195*	N.A.	0.497*	10 (6.1)	10 (7.7)	0 (0)	0.122*	12 (3.8)	0.029
Cefoxitin	1 (0.7)	0 (0)	0 (0)	1 (2.4)	N.A.	0.412*	0.467*	3 (1.8)	3 (2.3)	0 (0)	1.000*	4 (1.3)	0.624*
**Carbapenems**													
Meropenem	0 (0)	0 (0)	0 (0)	0 (0)	N.A.	N.A.	N.A.	1 (0.6)	1 (0.8)	0 (0)	1.000*	1 (0.3)	1.000*
**Quinolones**													
Nalidixic Acid	42 (28)	14 (29.2)	14 (23.3)	14 (33.3)	0.492	0.265	0.670	100 (60.6)	89 (68.5)	11 (31.4)	<0.001	142 (45.1)	<0.001
Ciprofloxacin	7 (4.7)	1 (2.1)	3 (5)	3 (7.1)	0.627*	0.688*	0.336	67 (40.6)	63 (48.5)	4 (11.4)	<0.001	74 (23.5)	<0.001

*P*-values: Chi-square test and confidence level of 95%, * Fisher exact test and confidence level of 95%.p^*a*^: babies and non-vendor adults, p^*b*^: babies and chicken vendors, p^*c*^: chicken vendors and non-vendor adults, p^*d*^: non-organic and organic chickens, p^*e*^: humans and chickens. ESBL, Detection of Extended Spectrum Beta-lactamases; N.A., not applicable.

#### Humans

Human isolates (*n* = 150) were obtained from babies aged 0–2 years (40%, *n* = 60), adult non-vendors (32%, *n* = 48), and chicken vendors in local markets (28%, *n* = 42). MDR isolates were more frequent in chicken vendors (38.1%, *n* = 16) compared to non-vendors (22.9%, *n* = 11). Isolates from chicken vendors presented higher resistance rates to florfenicol (16.7%, *n* = 7) compared to non-vendor adults (4.2%, *p* = 0.077, Fisher’s exact test) and babies (5%, *p* = 0.087, Fisher’s exact test). However, they were not more resistant to chloramphenicol (11.9 vs. 4.2%, *p* = 0.245, Fisher’s exact test). *E. coli* isolates from babies presented high resistance levels to tetracycline (45%, *n* = 27), trimethoprim/sulfamethoxazole (60%, *n* = 36), amoxicillin (65%, *n* = 39), azithromycin (13.3%, *n* = 8), chloramphenicol (15%, *n* = 9), cefalotin (15%, *n* = 9), cefotaxime (8.3%, *n* = 5), and gentamicin (3.3%, *n* = 2).

#### Chickens Versus Humans

Overall, resistance rates were higher among chicken *E. coli* compared to human isolates (Table 1), including MDR (63 vs. 37.3%, *p* < 0.001, Chi-square test) and ESBL-producing *E. coli* (30.9 vs. 4.0%, *p* < 0.001, Chi-square test). Additionally, we found higher florfenicol resistance in 43.1% (*n* = 71) of chicken isolates and 16.7% (*n* = 7) of chicken vendors compared to other groups. Further resistance results are shown in Table 1.

### Genomic Analysis

**FIGURE 2:**
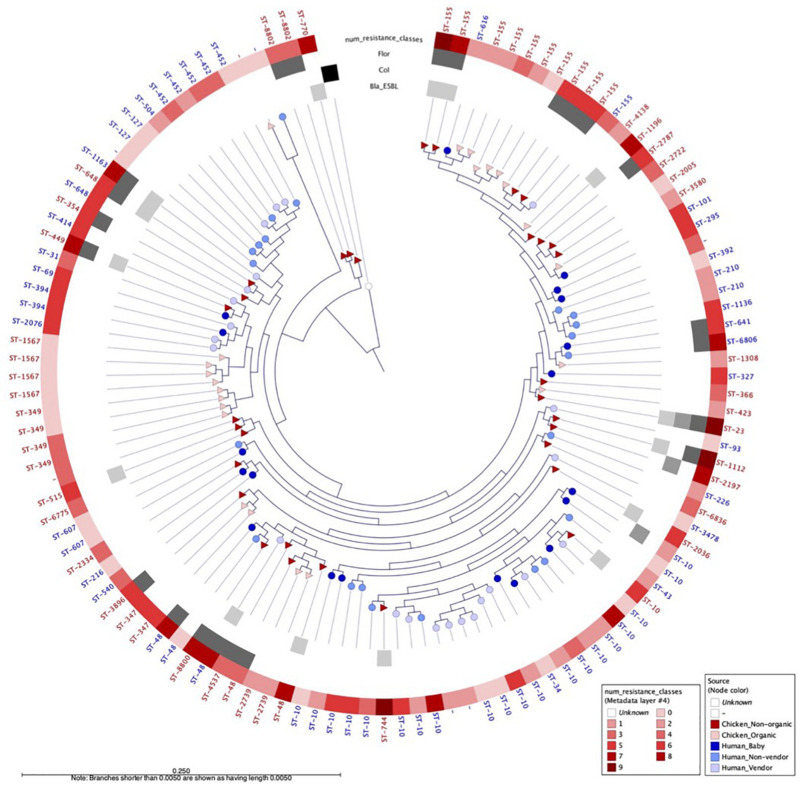
Maximum likelihood phylogenetic tree built from the alignment of 2,233 *E. coli* core genes from 118 human and animal isolates, using *E. fergusonii* as outgroup. Nodes are shaped by host type (circle = human, triangle = chicken) and colored by sampling group. Outer gray circles indicate presence of ESBL, *mcr-1*, and *floR* genes. Outer red circles indicate the number of antibiotic classes to which the isolate is resistant. WGS-based sequence type (ST) is indicated for each isolate.

**FIGURE 3:**
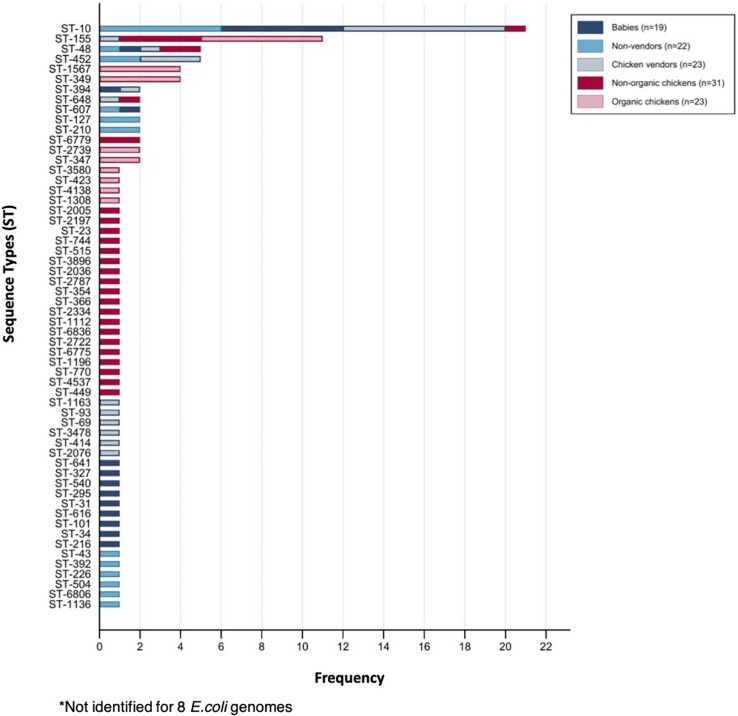
Sequence types (ST) identified among sequenced *E. coli* isolates.

We selected a random subset of 118 isolates from babies (*n* = 19), adults (*n* = 22), chicken vendors (*n* = 23), non-organic chickens (*n* = 31), and organic chickens (*n* = 23) to further understand the flow of *E. coli* phylogroups and ARGs between animals and humans. The genomic dataset had a mean N50 of 102,136 bp (SD = 49,584 bp) and a mean total length of 4,490,970 bp (SD = 1,222,343 bp). Pangenome analysis using Roary identified a core genome (i.e., genes found in ≥99% of isolates) of 2,304 genes and an accessory genome (found in ≤15% of isolates) of 26,135 genes. To assess the genomic similarity between isolates, we built a maximum-likelihood phylogenetic tree from the pangenome alignment (Figure 2) and calculated all pairwise SNP distances (Supplementary Figure 1). We identified 58 sequence types (ST) and 14 clonal complexes in the dataset (Figure 3). ST-10 (*n* = 21), ST-155 (*n* = 11), ST-48 (*n* = 5), and ST-648 (*n* = 2) were assigned to isolates of both animal and human origin. Highly similar isolates (differing in less than 100 SNPs across their pangenomes) were only found within host groups. STs shared by humans and chickens were more distantly related: ST-155 isolates (differing in 951 SNPs) were found in organic chickens and babies; ST-10 (1,046 SNPs), ST-155 (1,141 SNPs), ST-48 (1,542 SNPs), and ST-648 (13,470 SNPs) were shared by chicken vendors, non-vendors and market chickens.

**FIGURE 4:**
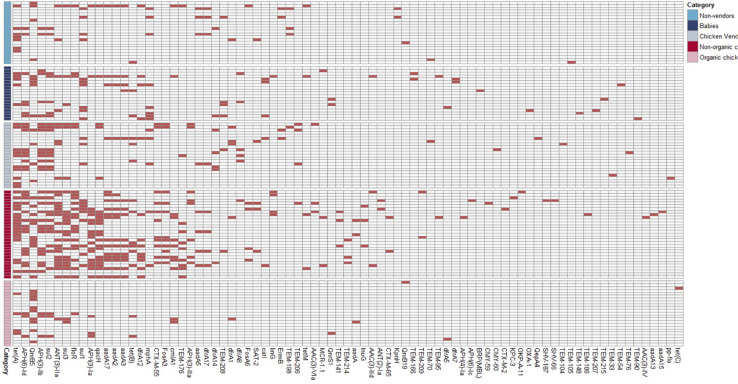
Antimicrobial resistance genes detected among sequenced *E. coli*.

**FIGURE 5:**
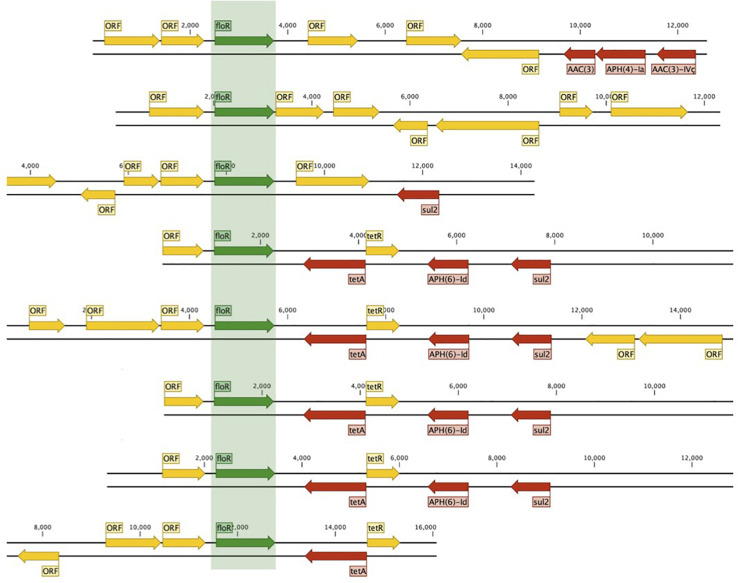
Mobilization of *floR* in conjugative plasmids from animals and humans. Eight unique (80% ID clustering) *floR*-containing plasmids were found in 22/118 humans and chicken isolates.

We identified 604 ARG hits and 81 unique ARGs in the dataset (Figure 4) with a mean of 5.1 genes (95%CI: 4.2–6.0) per isolate. Detected ARGs are associated with resistance to beta-lactams (*n* = 30), aminoglycosides (*n* = 18), trimethoprim (*n* = 7), amphenicols (*n* = 4), tetracyclines (*n* = 4), quinolones (*n* = 4), sulfonamides (*n* = 3), fosfomycins (*n* = 2), lincosamides (*n* = 2), macrolides (*n* = 1), glycopeptides (*n* = 1), polymyxins (*n* = 1), streptogramins (*n* = 1), and streptothricins (*n* = 1). Fifteen isolates (13 from market chickens and two from vendors) were positive for ESBLs; we found *blaCTX-M-55* in 73% (11/15) of them, in plasmid contigs that shared >96% sequence similarity between chickens and vendors. We found the *blaKPC-3* gene encoding carbapenem resistance in one market chicken isolate. Additionally, three isolates (two from market chickens and one from a baby) had the *mcr-1* colistin resistance gene (Figure 2). Forty-five plasmid replicon markers were identified in both humans and chickens (Supplementary Data Set 1 and Supplementary Figure 2). The most frequent markers were IncFIB (AP001918) (42.4%), Col (pHAD28) (35.6%), and IncFII (28%). Some markers were found in only one host type, such as IncB/O/K/Z_4 (*p* < 0.001) and Col156 (*p* = 0.003) in humans, and IncHI1B (*p* < 0.001) in chickens (Supplementary Figure 2). We did not find significant differences in plasmid markers among CTX-M, mcr-1, and blaKPC-3 producers from chickens and humans (Supplementary Table 3). We identified the *floR* gene in 18.6% (22/118) of genomes, and their contigs clustered into eight unique (>80% identity) sequences that matched to plasmid replicons of the IncF family (Figure 5). They shared a common theme in which *floR* was often found along with other antibiotic resistance genes (*tetA*, *APH(6)-Id*, *sul2*) and proteins predicted to be involved in horizontal gene transfer and DNA recombination (transposases, resolvases, recombinases, relaxases). This suggests that *floR* has been transferred on multiple occasions to MDR plasmids commonly shared by animal and human hosts. The *cmlA1* and *catA1* genes (which encode resistance to chloramphenicol but not florfenicol) were found in 11 and four genomes, respectively. A summary of the genomic analysis is described in Supplementary Data Set 2. All 604 ARG hits are listed in Supplementary Table 4.

## Discussion

We compared the resistance rates, genotype distributions, and ARGs present in commensal *E. coli* isolates from human and chicken populations. 315 *E. coli* isolates from humans (*n* = 150) and chickens (*n* = 165) were identified, with chickens showing higher rates of MDR (63 vs. 37.3%) and ESBL (30.9 vs. 4%) phenotypes. Poultry production is one of the largest and most widespread industries in Peru, making use of large quantities of various antimicrobials critical for human medicine (Page and Gautier, 2012; World Health Organization [WHO], 2017a). Despite their importance for treatment and disease prevention, their extended and unregulated use as growth promoters increases selective pressure for MDR bacteria (Diarra and Malouin, 2014). Our results highlight the potential consequences of this practice in poultry production.

Given that many LMICs are now transitioning to industrial models of animal production, there is a concern that extensive animal exposure to antibiotics may result in the “spillover” of resistant bacteria and ARGs into humans. Although ARG transfer has been extensively studied in pathogenic organisms, the vast majority of transfer events occur silently among non-pathogenic bacteria in host-associated and environmental microbial communities (Smillie et al., 2011; Pehrsson et al., 2016; Wang et al., 2017). *E. coli* and members of the *Enterobacteriaceae* are well adapted to the gut environment, acquiring diverse functions and ARGs to colonize their hosts (Szmolka and Nagy, 2013). It is thus likely that ARGs can accumulate in commensal strains to enrich the human gut resistome, and later be mobilized into pathogenic strains to become multidrug-resistant (Penders et al., 2013).

Increased global consumer awareness of how animal meat is produced has increasingly lead to the establishment of organic and free-range farms (Holtcamp, 2011). This production model aims to stop the widespread use of antibiotics as prophylactics and growth promoters in chickens under the premise that it will reduce AMR rates in exposed bacteria due to an absence of this selective pressure (Tang et al., 2017). The lower rates of AMR found in organic chickens compared to conventionally raised ones support this assertion. Furthermore, organic chicken isolates were entirely susceptible to gentamicin, amoxicillin with clavulanic acid, cefotaxime, cefepime, ceftazidime, and cefoxitin; the first three, together with florfenicol, are frequently found as active ingredients in local commercially available premixes aimed toward infection prevention and enhancement of growth performance.

The number of peri-urban communities has increased dramatically in recent decades in Peru and other LMICs, on par with poorly regulated neighborhood markets. Despite regulatory authorities’ supervision, many small markets function clandestinely for slaughtering to meet the consumers’ demand for “fresh” goods. Such consumer preferences, combined with other external factors, result in the poultry industry trading around 80% of its chicken production live (De León, 2009). Consequently, poultry butchering and handling practices in market stalls and related environments (including households) pose a risk of exposure to fecal cross-contamination from the viscera, a possible transfer route of animal-derived *E. coli* into the human gut. Despite their close contact with chickens and regular manipulation of viscera, *E. coli* isolates from chicken vendors did not fully match the resistance patterns observed in chicken isolates; this may be in part because the use of antibiotics to treat human infections also determine the resistance patterns of *E. coli* in the human gut. Shared STs (e.g., ST-10, ST-155, ST-48) were found in both chicken vendors and market chickens, coinciding with previous reports of globally successful STs linked to zoonotic transmission (Cohen Stuart et al., 2012; Yamaji et al., 2018; Falgenhauer et al., 2019; Hussain et al., 2019). However, shared STs differed in 900+ SNPs across their core genomes, which rules out a direct transmission between hosts and may reveal host-specific adaptations in *E. coli*.

Florfenicol, which is not approved for use in humans, was the only antibiotic tested for which resistance levels were significantly higher in chicken vendors than other human groups. We found florfenicol resistance in 43% of non-organic chicken isolates and 17% of chicken vendors. The *floR* gene was found in 17 *E. coli* genomes from chickens and five from humans and was associated with conjugative plasmids that were highly similar between humans and animal isolates (Figure 5). The high diversity of *floR*-carrying plasmids and the fact that they were identified in 15 different STs may reflect a strong selective pressure to maintain resistance to florfenicol in chicken *E. coli* populations. The *floR* gene confers resistance to florfenicol and chloramphenicol via an efflux pump mechanism (Bischoff et al., 2005; Braibant et al., 2005; Van Hoek et al., 2011) and is readily transferred among Gram-negative bacterial lineages via conjugative plasmids (Kruse and Sorum, 1994; Singer et al., 2004). We hypothesize that resistance to florfenicol in humans may occur via the colonization by *floR*-positive strains of animal origin or plasmid conjugation from animal strains into human commensals, both facilitated by improper handling of chicken meat by both vendors and consumers. This identifies *floR* as a potential marker of antibiotic resistance in humans that can be traced directly to antibiotic use in animals.

Resistance to last-resort drugs such as colistin and carbapenems is increasing worldwide (Peyclit et al., 2019). The *blaKPC-3* gene and phenotypic resistance to meropenem were observed in one market chicken isolate. The *bla*KPC-2 gene had been described in *Klebsiella pneumoniae* from Peruvian hospital settings (Horna et al., 2017; Roach et al., 2020) but this is, to our knowledge, the first report of KPC-3 in Peru; its origins and spread into animal populations warrant further study. Three isolates harboring the colistin resistance *mcr-1* gene were found in humans and chickens. Colistin is used to treat human infections caused by carbapenem-resistant bacteria (Nation et al., 2017) and *mcr-1* has already been reported in local *E. coli* and *K. pneumoniae* clinical isolates (Ugarte-Silva et al., 2018; Deshpande et al., 2019). The import and trade of colistin in veterinary products was banned in Peru in 2019 (Diario Oficial El Peruano, 2019) but they were still in use in poultry farms at the time of sampling.

Despite our initial assumption that babies would present lower rates of resistance compared to adults, they had similar resistance profiles to chicken vendors and had higher rates of phenotypic resistance to amoxicillin and trimethoprim/sulfamethoxazole than adults. This supports the findings of a previous study in Peru that found older age protective against resistance (Kalter et al., 2010). Children are prone to play in soils and have a higher risk of colonization with enteropathogens via the fecal-oral route (Marquis et al., 1990; Lietzau et al., 2007; Fuhrimann et al., 2016). The effect may be exacerbated in this community, where water and adequate sanitation are not available in all households. Surveys collected during an ongoing cohort study in VES (unpublished data) indicate that the most commonly used antibiotics in this group were amoxicillin and amoxicillin with clavulanic acid, followed by trimethoprim/sulfamethoxazole and erythromycin, consumed between the first 2 months up to 2 years at a rate of 3.8 courses per child-year (Nadimpalli et al., 2020). Predictably, 63.9% of the baby isolates in our study exhibited resistance to amoxicillin and 52.5% to trimethoprim/sulfamethoxazole. Other antibiotics administered to this group but with no evidence of resistance were cephalexin, clarithromycin, azithromycin, ciprofloxacin, and furazolidone. Community-level education campaigns on antibiotic awareness, combined with behavior change interventions, could help limit the transmission of ARGs and resistant bacteria to babies.

Many reports have identified high levels of AMR in food animals and retail meats in the United States (Davis et al., 2018; Liu et al., 2018), China (Liu et al., 2017; Wu et al., 2018; He et al., 2019), and Europe (Gelbíčová et al., 2019; Mellor et al., 2019). Other studies have assessed ARG dissemination between isolates of human, animal, and environmental origin in LMICs (Nadimpalli et al., 2019; Subbiah et al., 2020). Our study is innovative because we compared animals raised with and without antibiotics, along with humans with varying levels of exposure to chicken meat, and used WGS to identify resistant isolates and ARGs among human and animal populations within the same community. However, it presents limitations: (i) We focused exclusively on *E. coli*, and our results do not account for the effects in other commensal species nor the transfer of mobile genetic elements (MGEs) between them; (ii) We included only one isolate for each subject, so we were unable to assess within-host *E. coli* diversity; (iii) The timeline for our collection of human stool samples and chicken intestinal and cloacal isolates do not overlap for much of the study; (iv) Illumina-based sequencing generated short reads that made it challenging to reconstruct full plasmid sequences. The use of long-read sequencing should vastly improve assemblies and provide new insights into the exchange and recombination of mobile genetic elements between hosts.

There are very few studies that can clearly link antibiotic use on farms with antibiotic resistance in humans, in part because of the lack of national antibiotic consumption surveys on farms and the high degree of HGT that occurs in enterobacterial genomes (Smillie et al., 2011; Partridge et al., 2018). WHO’s 2017 Global Action Plan on AMR calls for strengthening national surveillance capacities (World Health Organization [WHO], 2017b). Surveillance data on antibiotic use and resistance rates in poultry may serve stakeholders to make evidence-based decisions and policies, as is the case with high-income countries. AMR surveillance studies conducted in South America are scarce compared to other LMICs (Bantar et al., 2000; García et al., 2012; Baker et al., 2017; Bazzo et al., 2018). As the antibiotic resistome expands through the accumulation of gene cassettes or novel plasmids, and with further ARG transfer from animals into commensal human strains, last-resort drugs such as colistin and carbapenems will become increasingly ineffective to combat pathogenic microorganisms.

This study highlights the potential dissemination of resistance genes in *Escherichia coli* from market chickens into human populations. Policy change is needed to curb the misuse of antibiotics in agriculture, which in the past has been successful at reducing the environmental burden of resistance without hurting the productivity of farmers (Aarestrup et al., 2010; Marshall and Levy, 2011). It is estimated that Peru will increase antimicrobial use in livestock by 160% from 2010 to 2030 (Van Boeckel et al., 2015). To offset this scenario, the National Multi-sectoral Action Plan to Combat Antimicrobial Resistance is set to provide a set of milestones involving regulations of antimicrobial use in food animals by 2021 (Ministerio de Salud [MINSA], 2019). We support the view that restricting non-therapeutic supplementation of antibiotics in animal feed and regulating the drug classes used to treat disease will help prevent the dissemination of AMR from animals into humans. Our research may serve as a baseline for future interventions aimed at limiting the spread of AMR in the environment.

## Data Availability Statement

The datasets presented in this study can be found in online repositories. The names of the repository/repositories and accession number(s) can be found below: https://www.ncbi.nlm.nih.gov/bioproject/PRJNA633873/, PRJNA633873.

## Ethics Statement

The studies involving human participants were reviewed and approved by the Comité Institucional de Ética en Investigacion, Universidad Peruana Cayetano Heredia. Written informed consent to participate in this study was provided by the participants’ legal guardian/next of kin.

## Author Contributions

PT, RG, DB, MM, and GS designed the study. MM, GS, AD-B, BA, LC, and MC collected the samples and conducted the experiments. CC-V, PT, GS, MH, MP, and AL analyzed the datasets. PT, GS, AD-B, and MM wrote the manuscript. RG, DB, AL, and MP reviewed the manuscript. All authors contributed to the article and approved the submitted version.

## Conflict of Interest

The authors declare that the research was conducted in the absence of any commercial or financial relationships that could be construed as a potential conflict of interest.

## Footnotes

1 http://enterobase.warwick.ac.uk/

2 https://pubmlst.org/

## Supplementary Material

Supplementary Figure 1 Pairwise SNP distances between all pairs of *E. coli* isolates.

Supplementary Figure 2 Plasmid replicon marker sequences among sequenced *E. coli*.

Supplementary Table 1 Location, type, and sample size of humans and chickens included in the study.

Supplementary Table 2 Antibiotics used for resistance profiling.

Supplementary Table 3 Plasmid markers and bivariate analysis of sequenced *E. coli* from chickens and humans. ^∗^Fisher exact test and confidence level of 95%.

Supplementary Table 4 Antimicrobial resistance genes hits among 118 sequenced *E. coli*.

Supplementary Data Set 1 Plasmid markers detection among 118 *E. coli* isolates.

Supplementary Data Set 2 Genomic analysis of 118 *E. coli* isolates.
