# Supplementary material for: Market Chickens as a Source of Antibiotic-Resistant Escherichia coli in a Peri-Urban Community in Lima, Peru
Source: Front Microbiol. 2021 Mar 2;12:635871. doi: 10.3389/fmicb.2021.635871 (PMC7961087; doi:10.3389/fmicb.2021.635871)
Supplement: Supplementary Figure 1 — Pairwise SNP distances between all pairs of E. coli isolates. [file Image_1.pdf]

Figure 1. Pairwise SNP distances between all pairs of *E.coli* isolates.

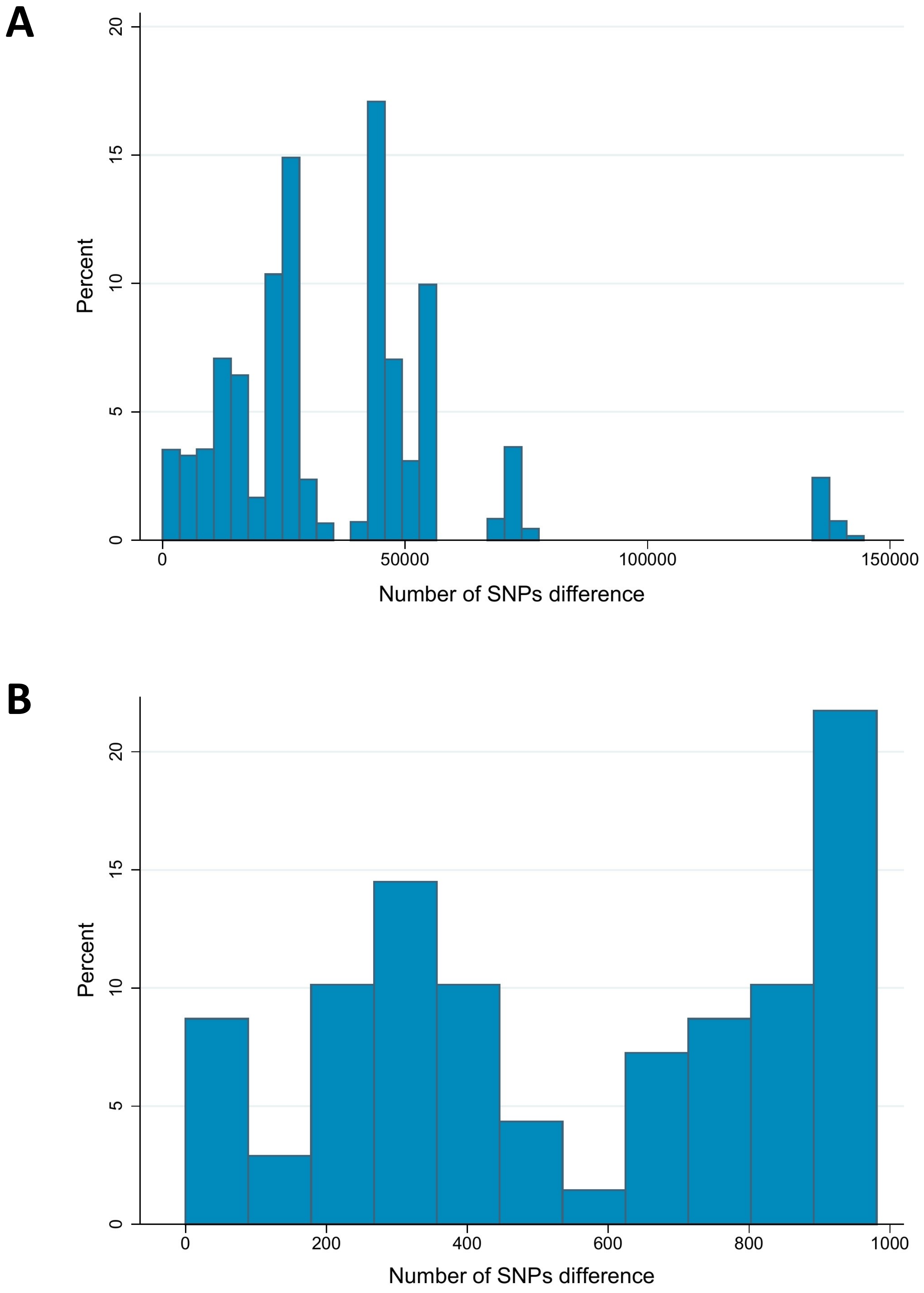

(A) Shows the full dataset, and (B) is part of the same figure drawn at a larger scale (less than 1000 SNPs difference) to show the smaller distances more clearly.
