# Supplementary figures and images for: Market Chickens as a Source of Antibiotic-Resistant Escherichia coli in a Peri-Urban Community in Lima, Peru
Source: Front Microbiol. 2021 Mar 2;12:635871. doi: 10.3389/fmicb.2021.635871 (PMC7961087; doi:10.3389/fmicb.2021.635871)

**Figure 2.** Plasmid replicon marker sequences among sequenced *E. coli*

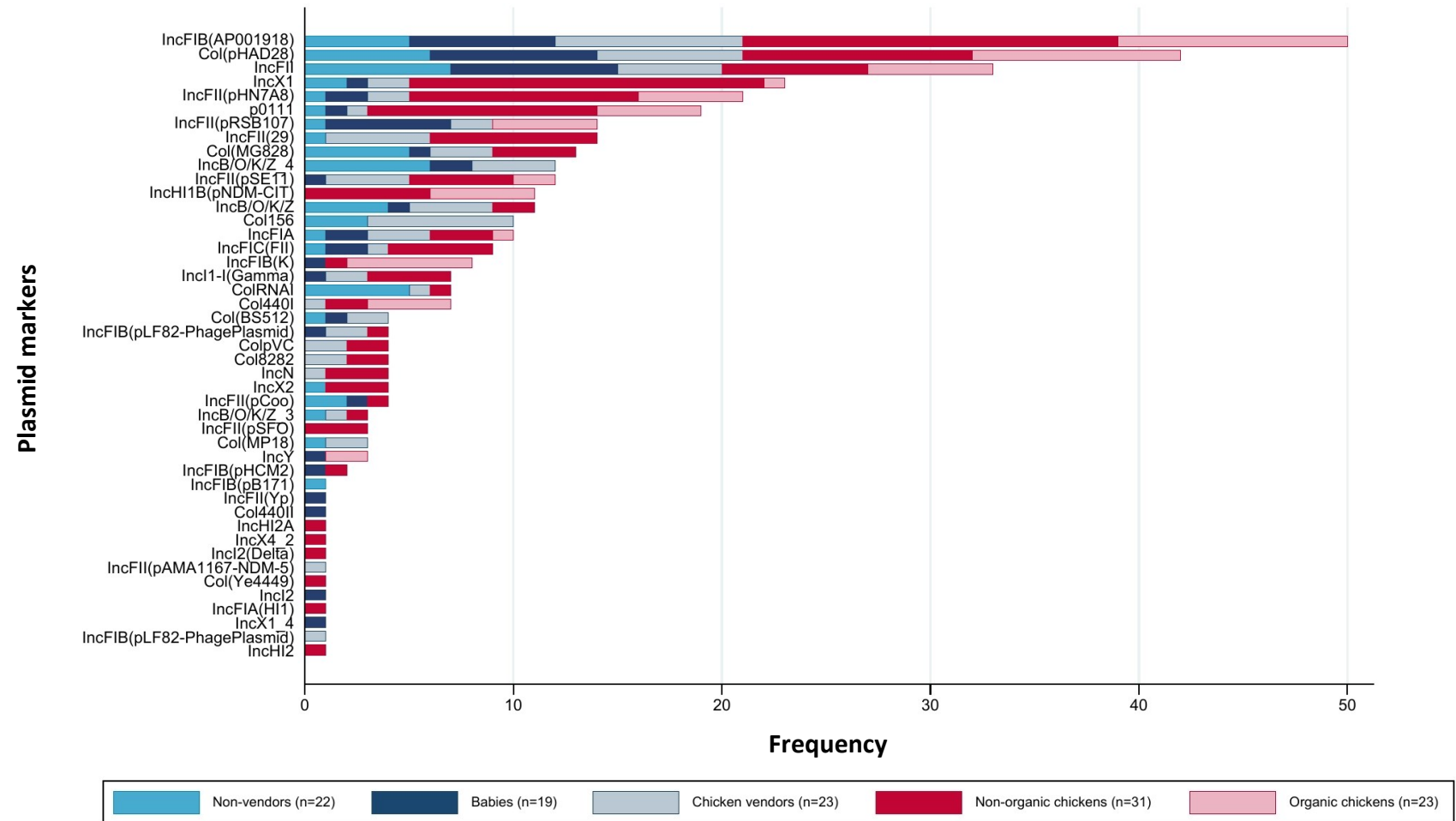

Supplement: Supplementary Figure 2 — Plasmid replicon marker sequences among sequenced E. coli. [file Image_2.pdf]
